# *RAS* and *BRAF* mutations in cell‐free DNA are predictive for outcome of cetuximab monotherapy in patients with tissue‐tested *RAS* wild‐type advanced colorectal cancer

**DOI:** 10.1002/1878-0261.12550

**Published:** 2019-09-30

**Authors:** Erik J. van Helden, Lindsay Angus, C. Willemien Menke‐van der Houven van Oordt, Daniëlle A. M. Heideman, Eline Boon, Suzanne C. van Es, Sandra A. Radema, Carla M. L. van Herpen, Derk Jan A. de Groot, Elisabeth G. E. de Vries, Maurice P. H. M. Jansen, Stefan Sleijfer, Henk M. W. Verheul

**Affiliations:** ^1^ Department of Medical Oncology Cancer Center Amsterdam Amsterdam UMC Vrije Universiteit Amsterdam The Netherlands; ^2^ Department of Medical Oncology Erasmus MC Cancer Institute Erasmus University Medical Center Rotterdam The Netherlands; ^3^ Department of Pathology Cancer Center Amsterdam Amsterdam UMC Vrije Universiteit Amsterdam The Netherlands; ^4^ Department of Medical Oncology Radboud University Medical Center Nijmegen The Netherlands; ^5^ Department of Medical Oncology University Medical Center Groningen The Netherlands

**Keywords:** biomarkers, *BRAF*, cell‐free DNA, cetuximab, colorectal cancer, *RAS* mutations

## Abstract

In metastatic colorectal cancer, *RAS* and *BRAF* mutations cause resistance to anti‐EGFR therapies, such as cetuximab. Heterogeneity in *RAS* and *BRAF* mutations might explain nonresponse in a subset of patients receiving cetuximab. Analyzing mutations in plasma‐derived circulating tumor DNA (ctDNA) could provide a more comprehensive overview of the mutational landscape as compared to analyses of primary and/or metastatic tumor tissue. Therefore, this prospective multicenter study followed 34 patients with metastatic colorectal cancer who were tissue‐tested as *RAS* wild‐type (exons 2–4) during routine work‐up and received third‐line cetuximab monotherapy. *BRAF* mutation status was also tested but did not exclude patients from therapy. At baseline and upon disease progression, cell‐free DNA (cfDNA) was isolated for targeted next‐generation sequencing (NGS). At 8 weeks, we determined that patients had benefited from treatment. NGS of cfDNA identified three patients with *RAS* mutations not detected in tumor tissue during routine work‐up. Another six patients had a *BRAF* or rare *RAS* mutation in ctDNA and/or tumor tissue. Relative to patients without mutations in *RAS/BRAF*, patients with mutations at baseline had shorter progression‐free survival [1.8 versus 4.9 months (*P* < 0.001)] and overall survival [3.1 versus 9.4 months (*P* = 0.001)]. In patients with clinical benefit (progressive disease after 8 weeks), ctDNA testing revealed previously undetected mutations in *RAS/BRAF* (71%) and *EGFR* (47%), which often emerged polyclonally. Our results indicate that baseline NGS of ctDNA can identify additional *RAS* mutation carriers, which could improve patient selection for anti‐EGFR therapies. Acquired resistance, in patients with initial treatment benefit, is mainly explained by polyclonal emergence of *RAS*,*BRAF,* and *EGFR* mutations in ctDNA.

**Abbreviations**
cfDNA: cell‐free DNA
CPCT: Center for Personalized Cancer Treatment
ctDNA: circulating tumor DNA
dPCR: digital polymerase chain reaction
EGFR: epidermal growth factor receptor
MAF: mutant allele frequency
MATV: metabolically active tumor volume
mCRC: metastatic colorectal cancer
MoAbs: monoclonal antibodies
NGS: next‐generation sequencing
OS: overall survival
PD: progressive disease
PFS: progression‐free survival
RT: room temperature

## 1 Introduction

Patients with metastatic colorectal cancer (mCRC), harboring *RAS* mutations, do not benefit from anti‐epidermal growth factor receptor (EGFR) monoclonal antibodies (MoAbs) such as cetuximab and panitumumab (Sorich *et al*., 2015). Despite patient selection for anti‐EGFR MoAbs based on *RAS* mutations in the tumor, only 40–45% of patients with wild‐type mCRC have clinical benefits resulting in partial response in 8–13% and stable disease in 32% of patients (van Helden *et al*., 2017; Karapetis *et al*., 2008; Lievre *et al*., 2008; Van Cutsem *et al*., 2015). Alternative biomarkers to predict treatment benefit are under investigation, including imaging of tumor uptake of cetuximab and early response evaluation with [^18^F]FDG PET, but have not led to clinical implementation so far (van Helden *et al*., 2016; Menke‐van der Houven van Oordt *et al*., 2015). In addition to *RAS* mutations, recent meta‐analyses demonstrated that *BRAF‐*mutated mCRC – which occurs in 8–10% of patients with *RAS* wild‐type mCRC – also fails to respond to anti‐EGFR MoAbs (Pietrantonio *et al*., 2015; Rowland *et al*., 2015). Consequently, patients with somatic *BRAF* p.V600E mutations are currently excluded from these therapies in clinical practice as well as in prospective clinical trials.

A potential explanation for the lack of response in patients with *RAS* and *BRAF* wild‐type tumors is the presence of intralesional and interlesional differences in mutational status. Although high concordance rates have been described in some studies (Vermaat *et al*., 2012), others do report heterogeneity in *RAS* and *BRAF* mutations ranging from 5% to 32% between the primary tumor and metastatic sites (Artale *et al*., 2008; He *et al*., 2016; Italiano *et al*., 2010; Kim *et al*., 2012; Vermaat *et al*., 2012). Tumor heterogeneity could result in missed *RAS‐* and *BRAF‐*mutated subclones, present under the detection limit of the assay or not present in the evaluated part of the tumors. In particular, the potential difference between primary tumor and metastatic site is of high relevance since in daily clinical practice primary tumor tissue is frequently being used to assess the mutational status of an individual's tumor, leaving mutations in metastatic cells undetected. This may result in nonresponse when a patient is treated in the metastatic setting.

Consequently, assessment of the mutational status of metastatic tissue prior to treatment with anti‐EGFR MoAbs is important. Although a biopsy from a metastatic lesion can be taken, this is a cumbersome procedure for patients and repetitive sampling is frequently not feasible. An alternative approach to identify the complexity and heterogeneity of all metastatic lesions in a minimally invasive manner is the analysis of plasma‐derived circulating tumor DNA (ctDNA) in cell‐free DNA (cfDNA), which consists of both healthy and tumor‐derived DNA. ctDNA comprises of short DNA fragments derived from tumor cells and theoretically represents the whole mutational landscape of all metastatic sites. Consequently, ctDNA might give a more accurate representation of the entire mutational profile than a single tumor tissue biopsy.

In untreated patients who started with anti‐EGFR blockade in combination with chemotherapy, it has been shown that oncogenic mutations as *KRAS* and *BRAF* can be detected in ctDNA (Misale *et al*., 2012). In addition, it has been described that mutations can appear in the circulation after acquired resistance in patients with initially wild‐type disease (Diaz *et al*., 2012; Thierry *et al*., 2014). However, most studies have described the mutational status in ctDNA by analyzing a limited number of genes and in patients treated with combination therapies of a chemotherapy backbone combined with cetuximab (Spindler *et al*., 2014; Thierry *et al*., 2017; Van Emburgh *et al*., 2016), which makes the interpretation of results with respect to anti‐EGFR MoAbs alone difficult.

In this prospective multicenter study, we report the mutational analyses of ctDNA in a unique cohort of 34 tissue‐tested *RAS* wild‐type [codon 12, 13 (exon 2); 59, 61 (exon 3); 117, 146 (exon 4)] mCRC patients treated with third‐line cetuximab monotherapy. Blood samples were collected prior to cetuximab therapy, during therapy and at disease progression. Mutations in ctDNA were measured by a large panel of 14 genes (236 hotspots), including *KRAS, NRAS, EGFR,* and *PIK3CA,* using a targeted next‐generation sequencing (NGS) approach with molecular barcoding. This approach allowed us to evaluate genetic profiles under the sole effect of cetuximab therapy. The aim of this study was to assess whether ctDNA could further improve patient selection for anti‐EGFR MoAb therapy. In addition, we aimed to gain more insight into the underlying mechanisms for acquired resistance to anti‐EGFR MoAb monotherapy.

## 2 Materials and methods

### 2.1 Study design and patients

The IMPACT‐CRC is a prospective phase I–II multicenter interventional study (registered with ClinicalTrials.gov, number NCT02117466) to evaluate the predictive value of [^89^Zr]cetuximab PET scans for cetuximab treatment response. As part of this study, plasma for cfDNA analyses was collected at baseline, after 2 weeks of treatment and at disease progression. All patients received cetuximab monotherapy as third‐line palliative systemic treatment. All 34 patients started with 500 mg·m^−2^ every other week. Based on the [^89^Zr]cetuximab PET/CT, eight patients received a higher dose cetuximab (750–1250 mg·m^−2^), whereas 26 patients continued with 500 mg·m^−2^ (E.J. van Helden, unpublished data). Patients were included in Amsterdam UMC, Vrije Universiteit Amsterdam, University Medical Center Groningen, and Radboud University Medical Center. The study was performed in accordance with the Declaration of Helsinki and approved by the Medical Research Ethics Committee of the Amsterdam UMC, Vrije Universiteit Amsterdam. All patients gave written informed consent prior to study procedures.

Patients were eligible for inclusion if they had unresectable *RAS* wild‐type metastatic colorectal cancer, had been treated with or had contra‐indications for standard chemotherapy (fluoropyrimidine, irinotecan, and oxaliplatin), and were naive for anti‐EGFR MoAbs. In all patients, mutational analysis was performed as part of routine clinical work‐up on either primary or metastatic tumor tissue and had to be *RAS* wild‐type. *RAS* wild‐type was defined as wild‐type in codons 12, 13 (exon 2); 59, 61 (exon 3); and 117, 146 (exon 4) of *KRAS* and *NRAS*. Patients with *BRAF* p.V600E mutations were allowed per protocol to participate, since only recently became clear that these patients do also not respond to anti‐EGFR MoAbs (Pietrantonio *et al*., 2015; Rowland *et al*., 2015).

Clinical outcome was defined as no clinical benefit for patients having progressive disease (PD) at 8 weeks and as clinical benefit for patients with stable disease or partial response according to RECISTv1.1 at 8 weeks (Eisenhauer *et al*., 2009). Additionally, progression‐free survival (PFS) and overall survival (OS) were evaluated, defined as the period between the first treatment cycle until PD or death, respectively. Patients that were still on treatment and/or alive at the last follow‐up date (December 1, 2017) were censored.

### 2.2 Plasma sample collection and handling

Prior to the first cetuximab cycle (baseline), after 2 weeks of treatment and at PD 18 mL of blood was drawn in Vacutainer^®^ EDTA tubes (BD, Franklin Lakes, NJ, USA). Plasma was isolated within 1 h after blood collection performing two sequential centrifugation steps: 820 ***g*** of 10 min at room temperature (RT) with brakes off, and 20 000 ***g*** for 10 min at RT. After centrifugation, plasma was snap‐frozen and stored at −80 °C until further handling.

### 2.3 Tumor tissue handling

According to standard of care, before start with cetuximab therapy, formalin‐fixed paraffin‐embedded material of the primary tumor and/or metastasis was tested for *RAS* (exon 2–4) and *BRAF* (exon 15) if the tumor percentage was ≥ 20% on hematoxylin eosin immunohistochemistry staining. For all patients included in the Amsterdam UMC, Vrije Universiteit Amsterdam, a TruSeq Amplicon Cancer Panel (TSACP; Illumina Inc, San Diego, CA, USA) was used as described previously (Sie *et al*., 2014). In case, tumor tissue was of insufficient quality for TSACP‐MiSeq‐NGS, and a high‐resolution melting technology‐based approach followed by direct sequencing to determine *RAS* and *BRAF* mutations was performed (Heideman *et al*., 2012; Kramer *et al*., 2009). For all patients included in University Medical Center Groningen and Radboud University Medical Center, multiplex PCR and PGM/Ion Torrent (Life Technologies, Carlsbad, CA, USA) sequence analyses were used as described previously (Boleij *et al*., 2015). Multicenter comparison of mutation testing for *RAS* and *BRAF* previously demonstrated an excellent reproducibility between these Dutch centers (Boleij *et al*., 2015).

In addition to routine work‐up, some patients underwent an additional biopsy prior to cetuximab therapy, which was analyzed via the Center for Personalized Cancer Treatment (CPCT; NCT01855477). This Dutch consortium offers next‐generation whole‐genome sequencing of snap‐frozen tumor material for the discovery of tumor mutations. To identify true somatic mutations, germline DNA collected from whole blood was sequenced in the same fashion as reference to tumor tissue (Bijlsma *et al*., 2016). The sequencing data of this CPCT biopsy came available after start of cetuximab therapy and did not influence clinical decision making.

### 2.4 cfDNA isolation and quantification

For cfDNA isolation, plasma samples were thawed and 4 mL of plasma was used. cfDNA isolation was performed for all 34 patients at baseline and 27 patients at disease progression. Additionally, for nine patients with clinical benefit, cfDNA was isolated from plasma collected after 2 weeks of treatment. cfDNA was isolated and eluted in 60 μL buffer using the QiaSymphony Circulating DNA kit (Qiagen, Venlo, the Netherlands) as per manufacturer's instructions and stored at −20 °C. CfDNA concentrations were quantified using the Quant‐iT dsDNA high‐sensitivity assay (Invitrogen, Life Technologies, Carlsbad, CA, USA) according to the manufacturer's instructions, and the Qubit fluorometer (Invitrogen) was used as read out.

### 2.5 Targeted NGS and digital PCR

A targeted NGS approach with molecular barcoding using Oncomine™ Colon cfDNA Assay (Thermo Fisher Scientific, Waltham, MA, USA) was applied for low limit (down to 0.1%) somatic variant detection according to the manufacturer's instructions. This assay consists of 14 colorectal cancer‐specific genes covering 236 hotspots and indels in 49 amplicons, including *AKT1, APC, BRAF, CTNNB1, EGFR, FBXW7, GNAS, HER2, KRAS, MAP2K1, NRAS, PIK3CA, SMAD4, and TP53*. CfDNA samples were thawed at RT and a maximum volume input of 13 μL of the cfDNA eluate was used, unless the amount of cfDNA in this volume exceeded an input of 20 ng cfDNA, and then, 20 ng cfDNA was used. This amount was used to standardize cfDNA input for targeted NGS between patients and allowed us to achieve a limit of detection of 0.1% (1 mutant copy in a background of 1000 wild‐type copies). Samples with cfDNA concentrations < 1·5 ng·μL^−1^ [33/69 (48%) samples] were concentrated using the Eppendorf™, Vacufuge™ Concentrator (Fisher Scientific). Baseline and PD samples originating from the same patient were sequenced within the same run. Analyses were done as previously reported, using Ion S5 XL sequencing system and 540 chips, and evaluated with a standard variant calling pipeline (Jansen *et al*., 2016). First, raw Ion S5 sequencing results with the Oncomine cfDNA assays were loaded into the TorrentSuite variant caller 5.6. Applying additional filtering, hotspot variants were called when at least 1000 unique molecules for that particular position were sequenced to achieve sufficient coverage for a limit of detection of 0.1% and if the mutant sequence was covered in three unique molecules and 10 reads (i.e., three reads per unique molecule).

Cell‐free DNA samples from two patients who harbored a *BRAF* p.V600E mutation in their tumor tissue and of whom the cfDNA analyses were negative according to targeted NGS (one sample failed during NGS, the other one tested wild‐type) were additionally tested for this mutation using a validated digital polymerase chain reaction (dPCR) assay (TaqMan^®^ SNP genotyping assays; Thermo Fisher Scientific), as described previously (van Dessel *et al*., 2017).

### 2.6 Tumor load

To compare the total measured cfDNA and ctDNA (mutant copies·mL^−1^ plasma) with the tumor burden in a patient, we evaluated tumor load on CT and [^18^F]FDG PET/CT scan. On the baseline diagnostic CT scan, the total number of metastases was evaluated per patient. Additionally, the sum of diameters of all tumor lesions was calculated.

Baseline [^18^F]FDG PET scan was performed within 2 weeks before the first treatment with cetuximab. The PET scans were created according to EANM guidelines (Boellaard *et al*., 2010). Briefly, patients fasted 6 h before tracer injection (target serum glucose ≤ 7 mmol·L^−1^). Mid‐femur‐skull vertex PET‐CT was performed 60 min (±5 min) after injection of [^18^F]FDG (3–4 MBq·kg^−1^) and combined with low‐dose CT (120 kVp, 50 mAs). PET data were normalized and corrected for scatter and randoms, attenuation, and decay. Tumor load on [^18^F]FDG PET scan is expressed as metabolically active tumor volume (MATV), which was calculated using a threshold of 50% of peak standard uptake value to define tumor volume.

### 2.7 Statistical methods

All statistical analyses were performed using ibm spss version 24 (IBM Corp, Armonk, NY, USA). A *P*‐value below 0.05 was used as cutoff for significance. To compare the presence of a mutation with treatment benefit, a Fisher's exact test was used. For survival analysis, patients without progression and patients that are still alive on December 1, 2017 were censored. Univariate analysis was done using Kaplan–Meier curves and Log Rank tests. With univariate and multivariate Cox regression, hazard ratios (HRs) were calculated (enter method). To correlate the concentration of ng cfDNA per mL plasma with the total volume of tumor load, a Spearman's ρ was used.

## 3 Results

### 3.1 Patients, plasma, and tumor tissue characteristics

In total, 34 patients were included from May 2014 until December 2016, and patient characteristics are described in Table 1. At the time of analyses (December 2017), all patients had progressed and 29 (85.3%) had died. Of all patients, 13 (38%) did not have treatment benefit. The median PFS of the whole cohort was 4.0 months (95% CI 2.7–5.2), and median OS was 9.0 months (95% CI 6.0–12.1).

**Table 1:** Baseline patient characteristics. SD, stable disease; PR, partial response

Characteristics	Clinical benefit (%)	No clinical benefit (%)	Total (%)
No. of patients	21 (62)	13 (38)	34 (100)
Median age (range)	64 (50–82)	64 (55–78)	64 (50–82)
Male gender	17 (81)	8 (62)	25 (73.5)
WHO performance status			
0	6 (28.6)	3 (23.1)	9 (26.5)
1	14 (66.7)	8 (61.5)	22 (64.7)
2	1 (4.8)	2 (15.4)	3 (8.8)
Primary tumor			
Right‐sided	1 (4.8)	8 (61.5)	9 (26.5)
Left‐sided	20 (95.2)	5 (38.5)	25 (73.5)
Previous treatments			
Fluoropyrimidine	21 (100)	13 (100)	34 (100)
Oxaliplatin	21 (100)	13 (100)	34 (100)
Irinotecan	18 (85.7)	13 (100)	31 (91.4)
Bevacizumab	15 (71.4)	8 (61.5)	23 (67.6)
Sunitinib	1 (4.8)	0	1 (2.9)
RECIST evaluation after 8 weeks			
PD	0	13 (100)	13 (38.2)
SD	18 (85.7)	0	18 (52.9)
PR	3 (14.3)	0	3 (8.8)
cfDNA			
Median cfDNA concentration in ng·mL^−1^ plasma (range)	46.5 (6.6–111)	54 (5.5–174)	49.4 (5.5–174)
*KRAS/BRAF* mutations	1 (4.8)	7 (53.8)	8 (23.5)
Median MATV on [^18^F] FDG PET (range)	148 (14–1189)	156 (40–805)	152 (14–1189)
PD at time of analysis	21 (100)	13 (100)	34 (100)
Deceased at time of analysis	16 (76.2)	13 (100)	29 (85.3)

### 3.2 Plasma isolation and raw analysis of samples

**Figure 1:**
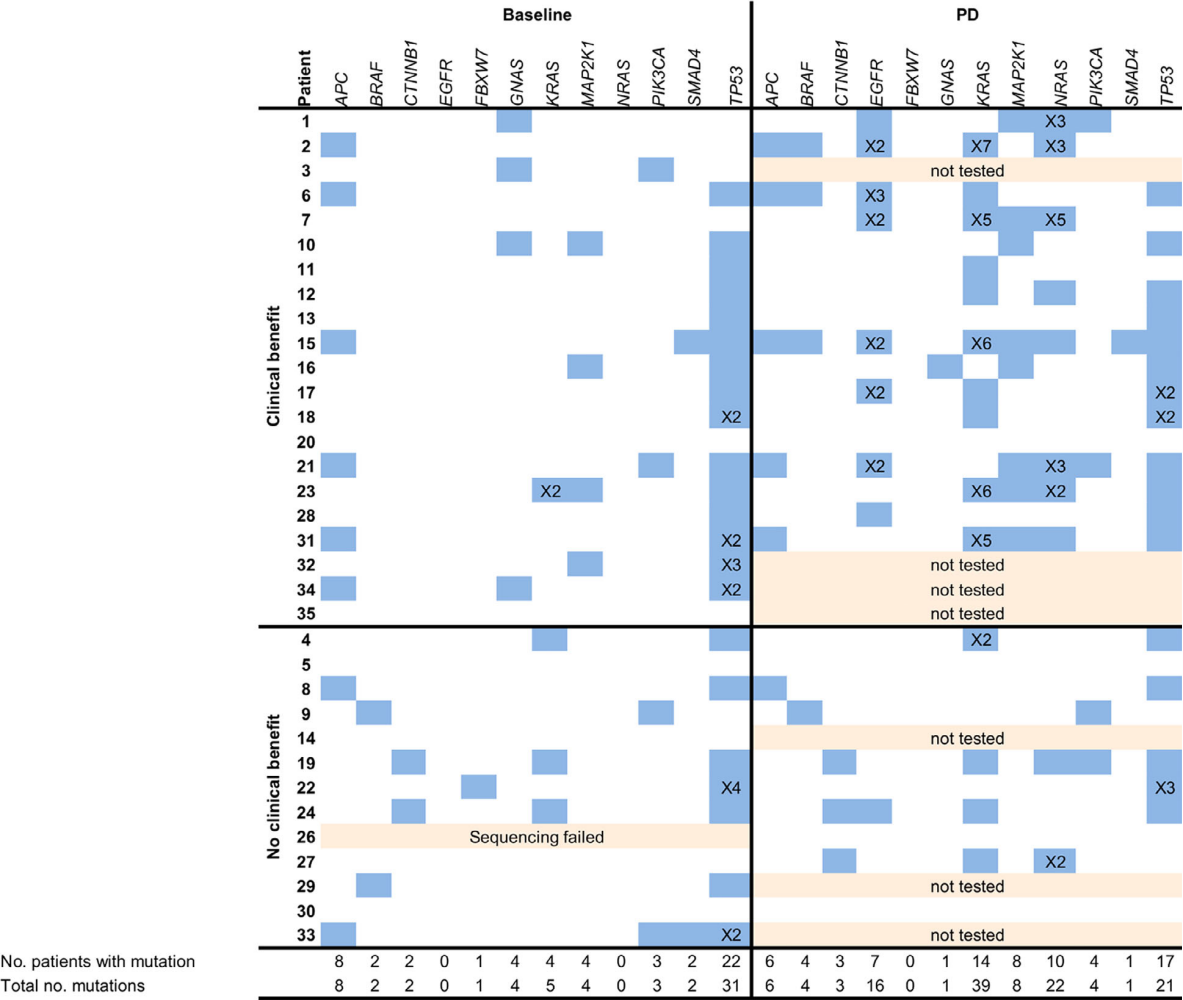
Comparison of mutational status as determined by ctDNA analyses at baseline and PD in patients with and without clinical benefit. The number behind the ‘X’ indicates the number of hotspot mutations within a gene.

The median cfNDA concentration at baseline was 49.4 ng·mL^−1^ plasma (range 5.5–784 ng·mL^−1^ plasma), and at PD 30.8 ng·mL^−1^ (range 4.91–228 ng·mL^−1^ plasma). A median of 20 ng (range 11.5–33.6 ng) was sequenced on the Ion S5 platform (Table S1). Variants were called based on our definition of a true positive (molecular coverage of ≥ 1000 and ≥ 10 mutant reads, and ≥ 3 mutated unique molecules). Five hotspots variants, which had a molecular coverage < 1000, were also considered true positives as these variants were detected in another sample collected at a different time point as well or if the hotspot was also detected in tumor tissue. The median molecular coverage of all amplicons was 2851 (range 0–20 000), and the median molecular coverage of mutated hotpots was 3436 (range 71–9641). In total, three samples failed during the sequencing process and were omitted from further analyses (Table S2). In summary, successful sequencing results were obtained from 33 of 34 baseline samples, from 7 of 9 2‐week samples, and all 26 samples at progression (Fig. 1).

### 3.3 *RAS* and *BRAF* mutations in tissue and ctDNA at baseline

#### 3.3.1 Tissue versus ctDNA

Sequencing results of baseline ctDNA obtained prior to start of cetuximab therapy were compared to the mutational status found in routinely tested tumor tissue (Table 2). In patients with treatment benefit (*n* = 21), no mutations in *KRAS*,* NRAS,* and *BRAF* were detected in tumor tissue. In ctDNA, however, a polyclonal mutation in codons 12 and 61 of *KRAS* was found in one patient (no. 23).

**Table 2:** Baseline mutations in genes: *BRAF, KRAS, NRAS*. Mutations detected in tumor tissue during routine work‐up and in cfDNA prior to start of cetuximab monotherapy. Mutations detected in tumor tissue and ctDNA are expressed in MAF

Genes	Nonresponders (*n *=* *13)			Responders (*n *=* *21)		
	Patient	Tissue (MAF%)	cfDNA (MAF%)	Patient	Tissue (MAF%)	cfDNA (MAF%)
*BRAF*	9	p.V600E (13)	p.V600E (1.97)		–	–
	14	p.V600E (29)	–			
	26	p.V600E (34)	p.V600E (6.6)^a^			
	29	p.V600E (44)	p.V600E (46.49)			
*KRAS*	4	–	p.G12A (1.34)	23	–	p.Q61H (0.38)
	19	–	p.Q61H (0.06)	23	–	p.G12A (0.15)
	24	p.G60D (43)	p.G60D (25.97)^b^			
	33	p.S89P (44)^c^	–			
*NRAS*		–	–		–	–

^a^NGS failed, *BRAF* p.V600E was detected by dPCR. ^b^This patient received cetuximab despite having a *KRAS* mutation, as mutations in codon 60 were not an exclusion criteria. ^c^ *KRAS* mutation detected by WGS, and this test result came available after treatment initiation. This hotspot is not covered by the Oncomine™ Colon cfDNA Assay.

In patients without treatment benefit (*n* = 13), four *BRAF* p.V600E mutations and one rare *KRAS* p.G60D mutation were detected in tumor tissue. Three of four *BRAF* p.V600E mutations were also detected in baseline ctDNA. In one patient (no. 26), sequencing of baseline ctDNA failed, but *BRAF* p.V600E status was assessed by a dPCR confirming the presence of the *BRAF* mutation at a mutant allele frequency (MAF) of 6.6%. In another patient (no. 14), the *BRAF* mutation was not detected in ctDNA by both sequencing and dPCR. No additional *BRAF* mutations over tumor tissue testing were identified in baseline ctDNA. The *KRAS* p.G60D mutation was confirmed in ctDNA, and two additional *KRAS* mutations were detected in ctDNA of patients 4 and 19, which were not detected in tumor tissue.

#### 3.3.2 Additional tissue analysis

For eight patients, mutational analyses were performed on two tumor tissue samples obtained prior to start of treatment (Table S3). Additional sequencing results came available after start of treatment and therefore did not influence clinical decision making.

In two patients, a *KRAS* mutation was found after an initially *RAS* wild‐type test. Both *KRAS* mutations were rare and not known as resistance‐inducing mutations, that is, codon 89 (*KRAS* p.S89P) and codon 60 (*KRAS* p.G60D). The first mutation was not covered by the initial *RAS* analysis; the latter was covered by the initial sequencing panel, but was not detected in the initial sample.

#### 3.3.3 *RAS/BRAF* mutations in ctDNA and tumor tissue are predictive for treatment response

**Figure 2:**
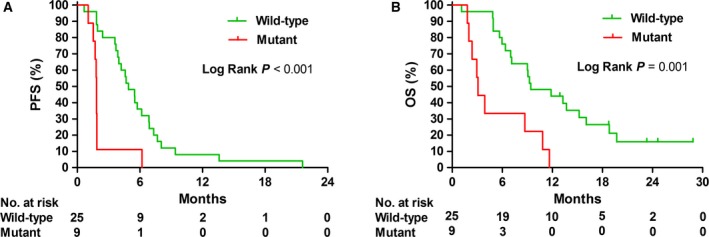
Progression‐free survival (A) and OS (B) for patients with *RAS* and/or *BRAF* mutations (mutant) versus patients without *RAS/BRAF* mutations (wild‐type) in tissue and ctDNA.

Patients with any *RAS/BRAF* mutations in either tumor tissue or ctDNA had less treatment benefit than patients who had a negative test result. Eight of 13 (61.5%) patients without clinical benefit had a *RAS/BRAF* mutation versus one out of 21 (4.8%) patients with clinical benefit (*P *=* *0.001). PFS was shorter for patients with *RAS*/*BRAF* mutations, with a median PFS of 1.8 months versus 4.9 months in wild‐type patients (*P *<* *0.001, HR 4.3; 95% CI 1.8–10.0, Fig. 2A). In multivariate analysis, correcting for WHO performance status (0 versus 1–2) and left versus right‐sidedness, any *RAS* or *BRAF* mutation remained correlated with PFS (*P *=* *0.004, HR 4.3; 95% CI 1.6–11.6). In line with PFS, OS was shorter in patients with *RAS‐*/*BRAF*‐mutated disease, with a median of 3.1 versus 9.4 months (*P *=* *0.001, HR 3.9; 95% CI 1.6–9.3, Fig. 2B). Also, with multivariate analysis, corrected for sidedness and WHO performance status, any *RAS*/*BRAF* mutation remained correlated with OS (*P *=* *0.007, HR 5.8; 95% CI 1.6–20.7).

### 3.4 Comparison of mutations in ctDNA: baseline, 2 weeks on treatment and at progressive disease

#### 3.4.1 ctDNA mutations at baseline versus 2 weeks

**Figure 3:**
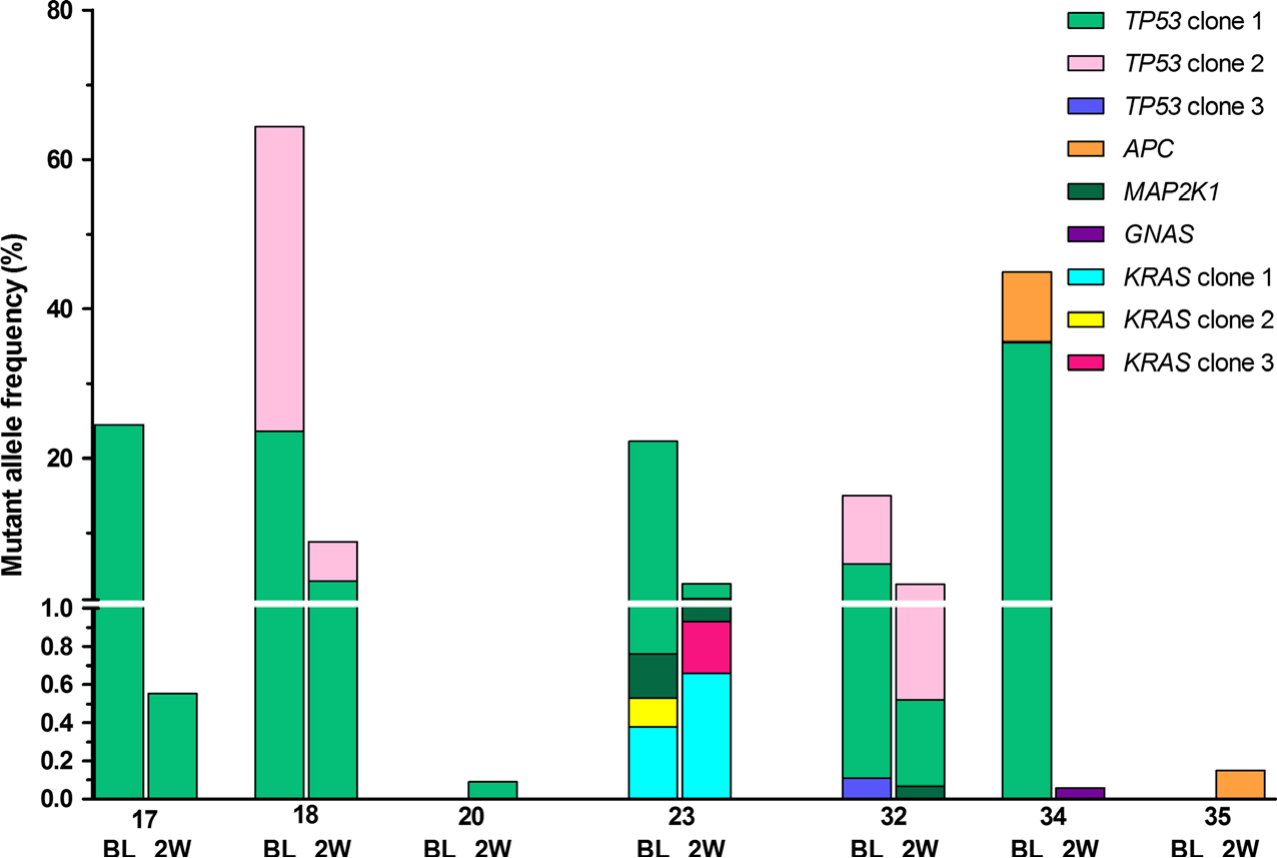
Paired baseline, 2‐week, and PD ctDNA‐sequencing results of patients with clinical benefit. Mutations were grouped per gene, and if patients harbored polyclonal mutations, the clones were numbered. For example, in patient 18 two *TP53* mutations were detected at baseline, clones 1 and 2, which both decreased in MAF at 2 weeks.

For nine patients with clinical benefit, plasma obtained after 2 weeks of treatment was available for cfDNA analyses. cfDNA concentrations decreased from a median of 44.7 ng·mL^−1^ plasma (range 13.3–784 ng·mL^−1^ plasma) at baseline to 18.9 ng·mL^−1^ plasma (range 7.4–41.7 ng·mL^−1^ plasma) after 2 weeks of cetuximab treatment (*P *=* *0.008), Fig. S1. Paired sequencing results showed that the MAF of dominant tumor clones present at baseline decreased after 2 weeks of treatment, suggesting a reduction in ctDNA load (Fig. 3). Detailed information on positions of mutations, MAF, and number of mutant molecules per mL plasma is available in Table S4.

#### 3.4.2 ctDNA mutations at baseline versus at progressive disease

To explore mechanisms of resistance, we compared the mutational signature at baseline and at disease progression. Paired cfDNA sequencing results were available for 17 patients with clinical benefit and eight patients without clinical benefit.

In 17 patients with initial clinical benefit, an evident increase in mutations in well‐known resistance‐inducing genes as *KRAS*,* NRAS,* and *BRAF* was observed at the time of progression [median sampling after 25 weeks (range 16–94 weeks)] (Fig. 1). Twelve patients (71%) had mutations in *KRAS* (*n* = 10) either or not combined with a mutation in *NRAS* (*n* = 8) and/or *BRAF* (*n* = 3) at disease progression. The total number of mutations in *KRAS* increased from 2 at baseline to 34 at PD, for *NRAS* from 0 to 19, and for *BRAF* from 0 to 3, respectively. Polyclonal *KRAS* mutations were present in one patient at baseline and in five patients at PD. Polyclonal mutations in *NRAS* were present in five patients at PD. For example, patient 23, who already harbored two *KRAS* mutations next to a dominant mutation in *TP53* at baseline (21%), showed a marked decrease in the dominant *TP53* mutation after 2 weeks (2%) of treatment and gained 4 *KRAS* and 2 *NRAS* mutations next to a clear increase in the *TP53* mutation (15%) at PD (Fig. S2).

**Figure 4:**
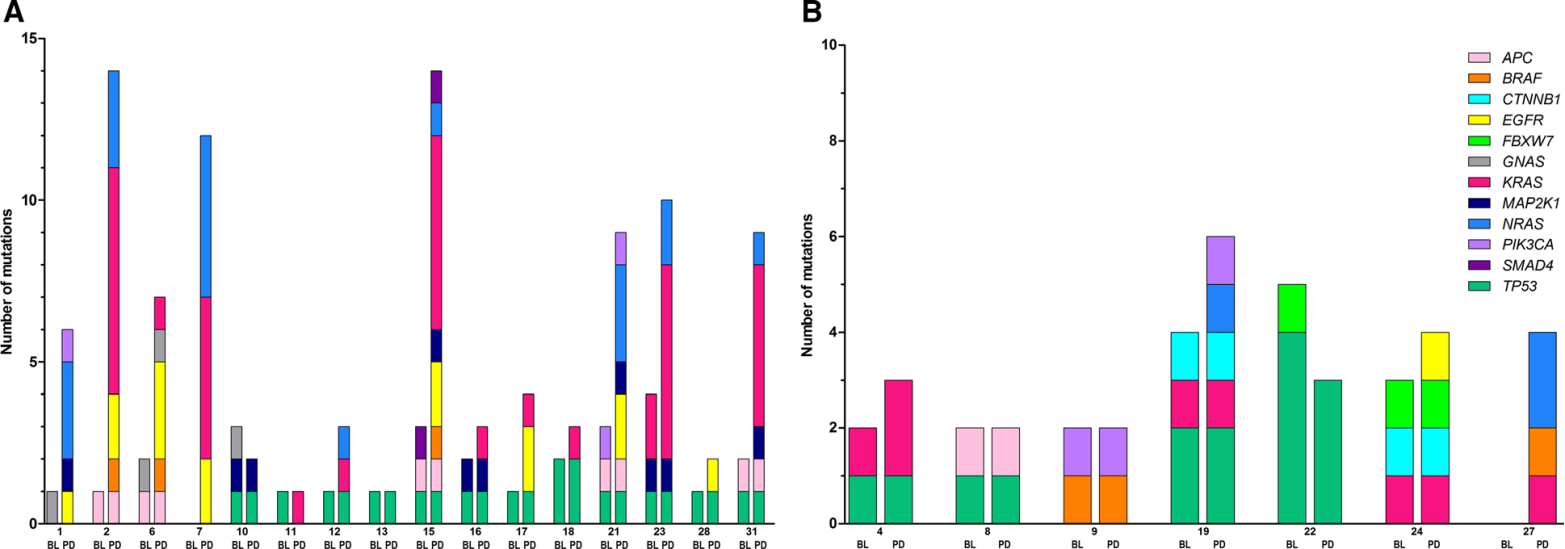
Paired baseline and PD ctDNA mutational analyses in patients with initial clinical benefit (A) and patients without clinical benefit (B). Mutations are depicted per gene, each gene having a separate color. Higher bars indicate polyclonal mutations. For example, patient 2 gained seven different KRAS hotspot mutations at disease progression. Patient 20 with clinical benefit and patient 30 without clinical benefit were not included in the graph because of the absence of mutations at baseline as well as PD.

In addition to the already established resistance‐inducing genes, the progression samples of patients with initial response to anti‐EGFR MoAbs were also enriched for *EGFR* mutations. Mutations in *EGFR* were detected in 8/17 (47%) patients at disease progression, which were not present at baseline, neither in ctDNA nor in tumor tissue. In 6/8 of patients with an *EGFR* mutation, polyclonal mutations occurred. These *EGFR* mutations were located in codons 464, 465, and 492, and code for the epitope binding site of cetuximab (Sickmier *et al*., 2016). In addition, the number of patients harboring *MAP2K1* mutations increased from four at baseline to eight at progression. Taken together, at disease progression 15/17 patients (88%) had a mutation related to anti‐EGFR MoAbs resistance (12 patients with *RAS* mutations, two patients with only *MAP2K1* mutations, and one patient with only an *EGFR* mutation). Mutated genes and the number of unique mutations per gene at baseline and PD are depicted in Fig. 4A.

In patients without clinical benefit, baseline, and PD (median sampling after 8 weeks, range 3–10 weeks), ctDNA mutation analyses demonstrated only a few differences (Fig. 1). Only one patient without baseline mutations in ctDNA nor tumor tissue gained mutations in *KRAS*,* NRAS,* and *BRAF* at progression in ctDNA. Patients 4, 19, and 24 gained all one additional mutation at progression: *KRAS, NRAS,* and *EGFR* mutations, respectively (Fig. 4B).

#### 3.4.3 Baseline ctDNA mutations: clinical benefit versus no clinical benefit

Baseline ctDNA of patients without clinical benefit was compared to baseline ctDNA of patients with initial clinical benefit to define whether there were differences in affected genes beyond *KRAS, NRAS,* and *BRAF* mutations*. APC*,* TP53*,* MAP2K1*,* SMAD4,* and *PIK3CA* mutations were present in baseline ctDNA samples of both patient groups. Mutations in *CTNNB1* were only present in baseline samples of two patients without treatment benefit. However, both *CTNNB1* mutations were present together with a *KRAS* mutation. *CTNNB1* is associated with constitutive RAF/MEK/ERK pathway activation (Malapelle *et al*., 2016). An overview of all mutations, in tissue and ctDNA from all time points, is shown in Table S4.

### 3.5 Left‐ versus right‐sided mCRC

Based on tissue‐tested mutation analyses, six out of nine patients with right‐sided mCRC had a *RAS* or *BRAF* mutation. Incorporating the ctDNA mutation analyses, eight out of nine patients with right‐sided mCRC had a *RAS* or *BRAF* mutation (*P *<* *0.001). The one patient with right‐sided mCRC without any *RAS* or *BRAF* mutation experienced treatment benefit, with disease control of almost 14 months and a censored OS of 23 months. Only one patient (1/25, 4%) with left‐sided mCRC had a polyclonal *KRAS* mutation in ctDNA analysis and was free of progression for 6.2 months and died 8.7 months after start of cetuximab therapy.

### 3.6 Tumor load versus cfDNA concentration

The sum of diameters of all metastases per patient did correlate to baseline cfDNA concentration (*P *=* *0.033), and also, the number of metastases (median 5.5 lesions, range 1–15) did correlate with cfDNA concentration (*P *=* *0.037). Moreover, the MATV on [^18^F]‐FDG PET highly correlated with baseline concentration cfDNA (ng cfDNA·mL^−1^ plasma) (Spearman's ρ 0.67, *P *<* *0.001; Fig. S3A). In addition, the total number of hotspot mutant molecules per mL plasma as a surrogate for mutational load also correlated with MATV on [^18^F] FDG PET (Spearman's ρ 0.50, *P *=* *0.003) (Fig. S3B).

## 4 Discussion

The results of the current study indicate that a subset of patients with *RAS* wild‐type tumors who have no clinical benefit on cetuximab monotherapy do have *KRAS* mutations in ctDNA. Our analysis of patients’ baseline ctDNA revealed three additional patients who had *KRAS* mutations (*KRAS* p.G12A, p.G61H, and a combination of the two) that had not been detected in tumor tissue. These discordant findings between tumor tissue and ctDNA are in line with previous reports that have demonstrated that mutations can be heterogeneous within primary tumor lesions, between synchronous lesions, and between metastases (Jeantet *et al*., 2016; Kosmidou *et al*., 2014; de Macedo *et al*., 2015; Normanno *et al*., 2015; Oltedal *et al*., 2011). Apart from such tumor heterogeneity, the sensitivity of sequencing assays used in tumor tissue testing could also have led to false‐negative results since most of the clinically used assays have a limit of detection of MAF > 5% (Shackelford *et al*., 2012). This hypothesis has recently been supported by Khan *et al*. (2018) who showed that *RAS* mutations in ctDNA could be confirmed in tumor tissue at low frequencies by using deep sequencing. The authors found that the MAFs of mutations detected in tumor tissue were indeed below the limit of detection of clinically used techniques. Furthermore, *KRAS* mutations detected in ctDNA at baseline were also detected at disease progression with higher MAFs, endorsing that *KRAS* is truly mutated in these cetuximab‐naive patients.

While most patients had known resistance‐inducing mutations, one patient harbored a rare *KRAS* p.G60D mutation in both tissue and ctDNA. Since this mutation was not in one of the codons known to be resistance‐inducing – and there has been anecdotal evidence of a patient with a p.G60D mutation having a partial response to cetuximab – this patient was allowed to participate in the study, but did not benefit from therapy (Molinari *et al*., 2011).

As this study included only those patients who had *KRAS* and *NRAS* wild‐type disease based on tumor tissue testing, a comparison of the mutational status in tissue versus ctDNA was not plausible for these genes. Since we included patients with *BRAF* mutations, a comparison of tissue versus ctDNA was possible in our cohort. We detected *BRAF* p.V600E mutations in ctDNA of three patients, in two patients by sequencing and in one by dPCR, and these *BRAF* mutations were also present in tumor tissue. One *BRAF* p.V600E mutation was present in tumor tissue of a fourth patient but was not detected in ctDNA with targeted NGS nor with an orthogonal technique as dPCR. We suggest three possible reasons for this. First, the molecular coverage of *BRAF* in the NGS experiment for this patient (patient 14) was 709 molecules. This is far lower than the median molecular coverage of *BRAF* of 2191 molecules that we measured in 67 samples, which might explain why this variant was not detected. A second possible explanation is that following surgical removal of the primary tumor that provided tissue for the test, subsequent metastases originated from a different clone that did not carry the *BRAF* mutation. Third, the cfDNA concentration of this patient was low, only 21.9 ng cfDNA·mL^−1^ plasma, which is much lower than the median baseline cfDNA concentration in our cohort (49.4 ng·mL^−1^ plasma). Since baseline cfDNA concentration was correlated with tumor load, low cfDNA concentrations could hypothetically lead to false‐negative results due to the fact the amount of tumor DNA carrying the mutation present in the circulation is simply too low. Nevertheless, for three out of four patients with the mutation in tumor tissue, the *BRAF* mutation was also detected in ctDNA. Although caution is warranted given the small number of patients, a detection rate of 75% is in line with that found in a previous study in non‐small‐cell lung cancer patients: This study compared the detection of the *EGFR* p.T790M mutation in ctDNA with that in tumor and reported a sensitivity of 70% (Oxnard *et al*., 2016).

While almost all patients with additional *KRAS* or *BRAF* mutations were resistant to therapy, we also had one patient with clinical benefit who nevertheless had a polyclonal *KRAS* mutation (p.G61H and p.G12A) in ctDNA, for which we suggest three potential explanations. First, this patient received a cetuximab dose escalation from 500 to 1250 mg·m^−2^, dosed every other week, based on the results of the [^89^Zr]cetuximab PET scan, which showed no uptake after one cycle of cetuximab (E.J. van Helden, unpublished data). Second, stable disease could also be a result of tumor heterogeneity, whereby only a small fraction of tumor cells harbor *KRAS* mutations and the majority are *RAS* wild‐type (Benvenuti *et al*., 2007; Karapetis *et al*., 2008). A final possible explanation is that there were other reasons for an indolent disease course regardless of treatment with cetuximab.

Given that the *KRAS* and *BRAF* mutations detected in ctDNA indeed conferring resistance to cetuximab, we were interested to see whether these mutations would be present throughout disease course and whether new mutations would appear. When we analyzed the mutation status in ctDNA at progression, we found that in patients who had shown initial treatment benefit, 12/17 (71%) patients had new *RAS* and/or *BRAF* mutations that were not detected at the start of the study. The fact that nine of these patients (9/12, 75%) had multiple mutations in these genes and codons suggests that the resistance to anti‐EGFR treatment is caused by the emergence of various clones harboring different mutations. Our finding of a relatively high number of patients treated with cetuximab who harbor *RAS* mutations at disease progression is in line with that of a previous study (Diaz *et al*., 2012; Misale *et al*., 2012; Siravegna *et al*., 2015; Van Emburgh *et al*., 2016). They reported *RAS* mutations in tumor tissue and ctDNA in 74% of patients who were mainly being treated with a combination of cetuximab and irinotecan. These mutations are most likely acquired by the tumor as a means of escape from the continuous pressure exerted by anti‐EGFR MoAbs. But it is also possible that the mutations are due to tumor heterogeneity resulting in the selection and outgrowth of multiple‐resistant *RAS*/*BRAF*‐mutated subclones, which are below the limit of detection at baseline.

Interestingly, at progression 8/17 patients (47%) with initial benefit had gained an *EGFR* mutation in ctDNA, and for six of these patients, these mutations were also polyclonal. *EGFR* mutations in codon 465 were detected in seven patients, in codon 464 in six patients, and in codon 492 in two patients. All of these *EGFR* mutations are located in domain III of the receptor and alter the epitope to which cetuximab binds, thereby inhibiting binding of cetuximab to EGFR (Arena *et al*., 2015, 2016; Bertotti *et al*., 2015; Esposito *et al*., 2013; Sickmier *et al*., 2016; Voigt *et al*., 2012). Esposito *et al*. (2013) have suggested that these mutations only occur after treatment with cetuximab, as evidenced by their study of 505 patients, in which mutations in tumor tissue were detected after anti‐EGFR therapy but not before. In our cohort, these *EGFR* mutations were also exclusively found at progression, rendering this mutation unsuitable for patient selection. It has been proposed that while these *EGFR* mutations occur after cetuximab therapy, they do not emerge after panitumumab therapy, leaving these tumor cells sensitive to panitumumab therapy (Montagut *et al*., 2012). However, given our observation that these mutations are almost always accompanied by other *RAS* or *BRAF* mutations, a treatment switch to panitumumab in *EGFR‐*mutated patients will probably not result in treatment benefit. Also, given the heterogeneity and convergence of the mutational pattern at progression, targeted blockage of the EGFR pathway will likely be difficult.

Finally, it is worth pointing out our finding of a correlation between the number of mutated molecules per mL plasma and the MATV measured by [^18^F]FDG PET before treatment. A similar correlation has been described previously in patients with non‐small‐cell lung cancer starting with erlotinib in a palliative setting (Winther‐Larsen *et al*., 2017). To our knowledge, our study is the first to show a similar correlation between the number of mutated molecules and MATV measured by [^18^F]FDG PET in patients with mCRC. Our study thereby supports the hypothesis that the total number of mutated molecules per mL plasma could serve as a surrogate for tumor load, which has also been described using CT to estimate tumor burden (Diehl *et al*., 2008). Important to note is that both techniques, [^18^F]FDG PET and ctDNA, are sensitive‐limited technologies hampering both techniques to detect low tumor burden. Next to the correlation between mutant molecules and MATV, we also found a correlation between the cfDNA concentration and MATV. It should be noted that the correlation between cfDNA and MATV might be less tumor specific, since cfDNA is composed of a small fraction of tumor DNA, while the majority is derived from normal apoptotic tissue and hematological cells (Elshimali *et al*., 2013; Jahr *et al*., 2001).

There are several limitations of our study including the small sample size. Second, in our study, tumor tissues were sequenced with panels used in daily routine practice. Therefore, comparative analyses of ctDNA and tumor tissue were hampered by the use of different techniques.

## 5 Conclusions

NGS of ctDNA in patients with tissue‐tested *RAS* wild‐type mCRC – tested as part of routine clinical work‐up – can identify additional *RAS* mutation carriers. The majority of patients with initial clinical benefit from cetuximab therapy gain mutations in genes such as *RAS, BRAF, and EGFR*, frequently occurring in multiple clones within individual patients. Hence, ctDNA analysis is a promising tool to optimize patient selection for anti‐EGFR monoclonal antibodies and a minimally invasive method to gain more insight in mechanisms accounting for resistance.

## Conflict of interest

DAMH has minority stake in Self‐screen B.V., a spin‐off company of VU University Medical Center Amsterdam (currently known as Amsterdam UMC, Vrije Universiteit Amsterdam), has been on the speaker′s bureau of Qiagen, and serves occasionally on the scientific advisory board of Pfizer and Bristol‐Meyer Squibb. L. Angus has attended the scientific advisory board of Merck BV once. All other authors declare no potential conflicts to interest (EJH, CWMHO, EB, SCE, SAR, CMLH, DJAG, EGEV, MPHMJ, SS, HMWV).

## Author contributions

EJH, CWMHO, EGEV, and HMWV involved in the conception and design. EJH, and LA involved in the analysis and interpretation of data (e.g., statistical analysis, biostatistics, computational analysis). EJH, LA, CWMHO, DAMH, EB, SCE, SAR, CMLH, DJAG, EGEV, MPHMJ, SS, and HMWV wrote, reviewed, and/or revised the manuscript. EJH, LA, and MPHMJ involved in the administrative, technical, or material support (i.e., reporting or organizing data, constructing databases). HWMV supervised the study.

## Supporting information


**Fig. S1.** cfDNA concentration measured in matched baseline, 2 weeks and PD samples. Each line indicates one patient. cfDNA concentrations were available for 9 matched baseline and 2 week samples, and for 6 PD samples.* Related samples Wilcoxon signed‐rank test.

**Fig. S2.** Patient 23 having a polyclonal *KRAS* mutation present at baseline, a marked decrease in the *TP53* p.R158H mutant allele frequency (MAF) after two weeks of treatment and an increase of the AMF at disease progression accompanied by emergence of four additional *KRAS* and two *NRAS* mutation.

**Fig. S3.** Scatter plot of the concentration cfDNA (in ng per mL plasma) (A) and the number of mutant molecules per mL plasma (B) versus the sum of metabolically active tumor volume (MATV) on [18F] FDG PET scan per patient.

**Table S1.** cfDNA concentrations and ng DNA input for targeted NGS.

**Table S2.** Sequencing failures.

**Table S3.** Double biopsies.

**Table S4.** Overview of all available mutation data.

## Data Availability

Raw data are available upon request. Please contact the corresponding author for details.
