# Supplementary figures and images for: RAS and BRAF mutations in cell‐free DNA are predictive for outcome of cetuximab monotherapy in patients with tissue‐tested RAS wild‐type advanced colorectal cancer
Source: Mol Oncol. 2019 Sep 30;13(11):2361–74. doi: 10.1002/1878-0261.12550 (PMC6822250; doi:10.1002/1878-0261.12550)

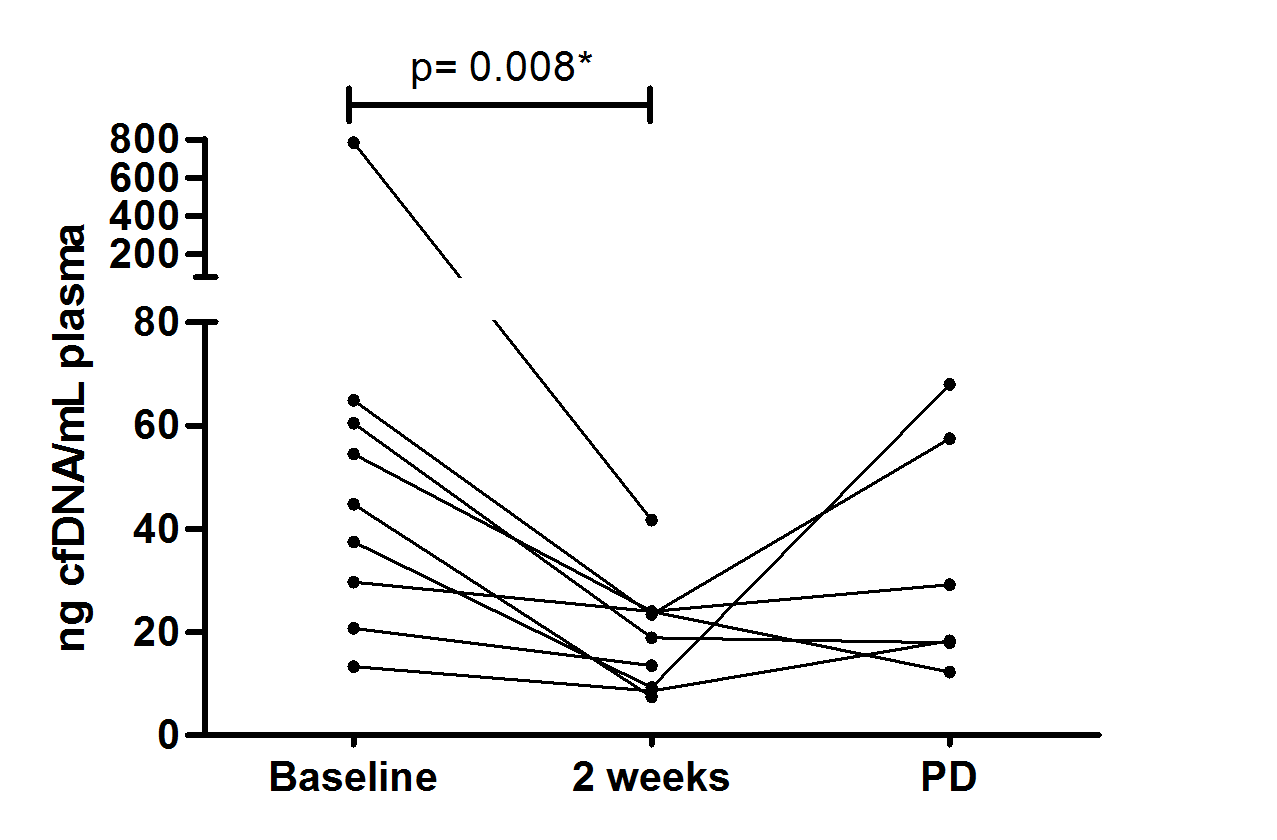

Supplement: Supplementary file 1 — Fig. S1. cfDNA concentration measured in matched baseline, 2 weeks and PD samples. Each line indicates one patient. cfDNA concentrations were available for 9 matched baseline and 2 week samples, and for 6 PD samples.* Related samples Wilcoxon signed‐rank test. [file MOL2-13-2361-s001.tif]

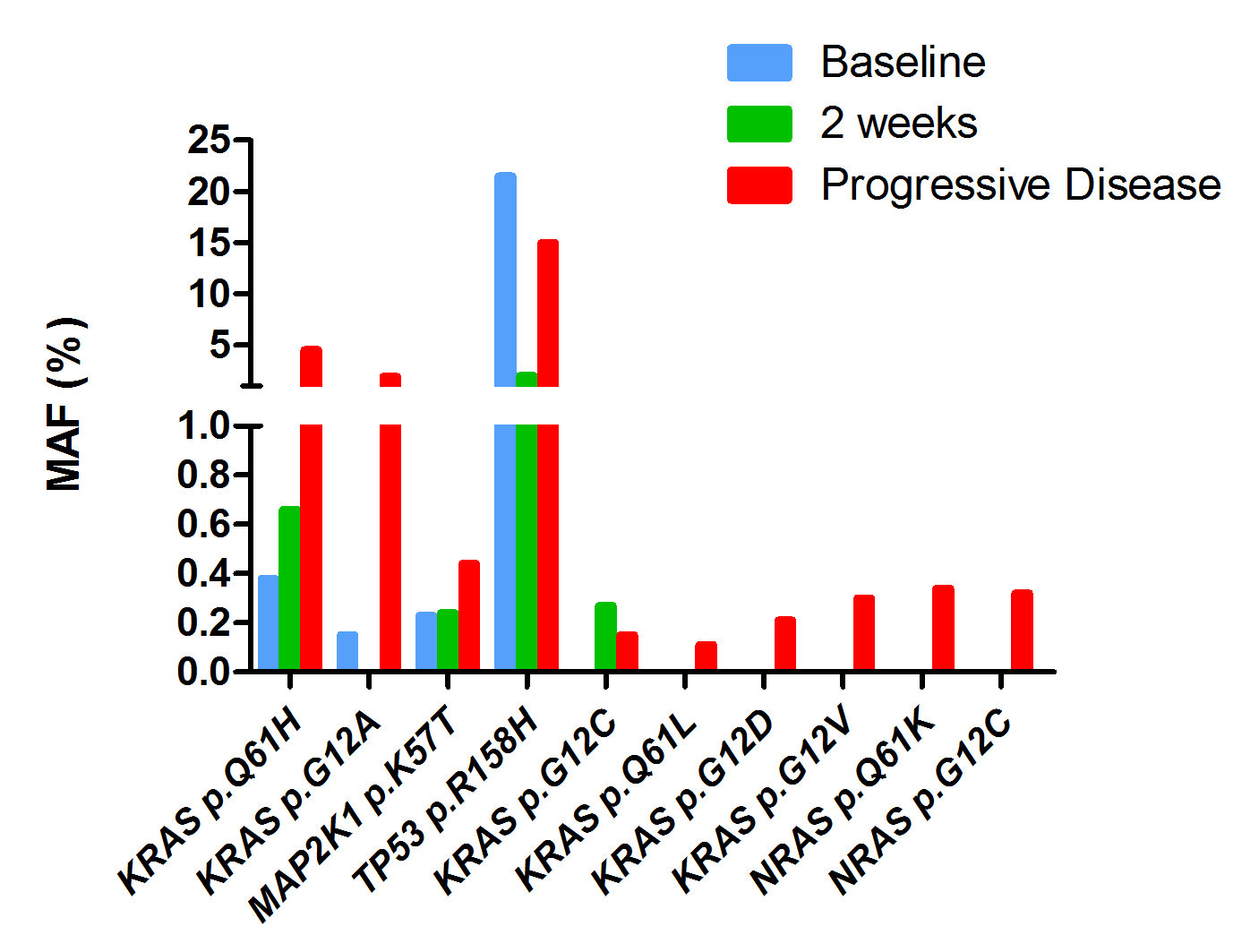

Supplement: Supplementary file 2 — Fig. S2. Patient 23 having a polyclonal KRAS mutation present at baseline, a marked decrease in the TP53 p.R158H mutant allele frequency (MAF) after two weeks of treatment and an increase of the AMF at disease progression accompanied by emergence of four additional KRAS and two NRAS mutation. [file MOL2-13-2361-s002.tif]

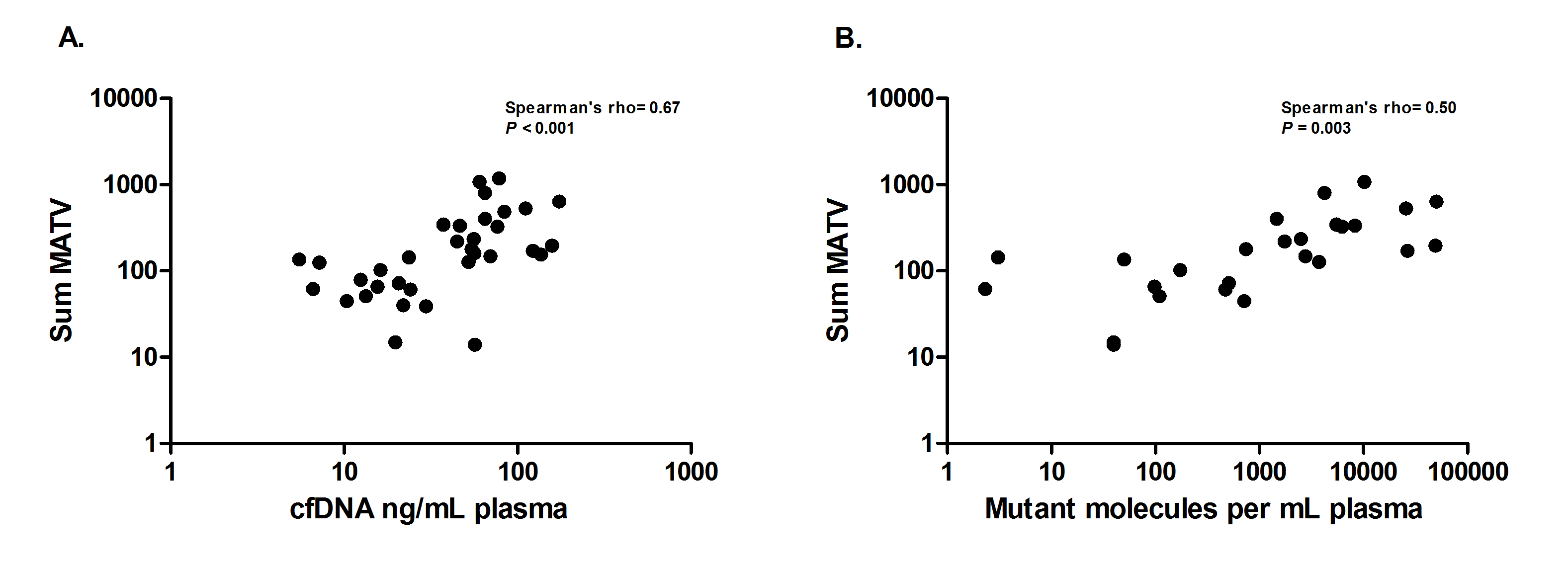

Supplement: Supplementary file 3 — Fig. S3. Scatter plot of the concentration cfDNA (in ng per mL plasma) (A) and the number of mutant molecules per mL plasma (B) versus the sum of metabolically active tumor volume (MATV) on [18F] FDG PET scan per patient. [file MOL2-13-2361-s003.tif]
